# World-first report of low anterior resection for rectal cancer with the hinotori™ Surgical Robot System: a case report

**DOI:** 10.1186/s40792-023-01705-9

**Published:** 2023-09-05

**Authors:** Ryo Miura, Koichi Okuya, Emi Akizuki, Masaaki Miyo, Ai Noda, Masayuki Ishii, Momoko Ichihara, Takahiro Korai, Maho Toyota, Tatsuya Ito, Tadashi Ogawa, Akina Kimura, Ichiro Takemasa

**Affiliations:** Department of Surgery, Surgical Oncology and Science, Sapporo Medical University School of Medicine, 291 Minami-1-jo Nishi 16-chome, Chuo-ku, Sapporo, Hokkaido, 060-8543 Japan

**Keywords:** Hinotori, Rectal cancer, Robotic surgery, Low anterior resection

## Abstract

**Background:**

The hinotori™ Surgical Robot System was approved for use in colorectal cancer surgery in Japan in 2022. This robot has advantages, such as an operation arm with eight axes, an adjustable arm base, and a flexible three-dimensional viewer, and is expected to be utilized in rectal cancer surgery. Herein, we report the world's first surgery for rectal cancer using the hinotori™ Surgical Robot System.

**Case presentation:**

A 71-year-old woman presented to our hospital with bloody stools. A colonoscopy revealed type 2 advanced cancer in the rectum, and a histological examination exposed a well-differentiated adenocarcinoma. Abdominal enhanced computed tomography divulged rectal wall thickening without significant swelling of the lymph nodes or distant metastasis. Pelvic magnetic resonance imaging showed tumor invasion beyond the intrinsic rectal muscle layer. The patient was diagnosed with cStage IIa (cT3N0M0) rectal cancer and underwent low anterior resection using the hinotori™ Surgical Robot System. Based on an adequate simulation, surgery was safely performed with appropriate port placement and arm base-angle adjustment. The operating time was 262 min, with a cockpit time of 134 min. Subsequently, the patient was discharged 10 days postoperatively without complications. The pathological diagnosis was pStage IIA (cT3N0M0) and the circumferential resection margin was 6 mm.

**Conclusions:**

We report the first case of low anterior resection for rectal cancer using the hinotori™ Surgical Robot System, in which a safe and appropriate oncological surgery was performed.

## Background

In recent years, surgical treatments have become more advanced and minimally invasive. Surgical robots have played a central role in this trend. Surgical robots were developed in the 1980s and their usage spread in the 2000s, particularly in Europe and the United States. The da Vinci Surgical System (Intuitive Surgical Inc., Sunnyvale, CA, USA) is the leading surgical robot used worldwide. Although the da Vinci Surgical System has a monopoly on the market, several companies have recently developed better surgical robots, and newer models are being commercialized. The introduction of robotic surgery is progressing rapidly in Japan. The country currently ranks second worldwide and first in Asia in terms of the number of surgical robots in its fleet. However, surgical robots are expensive, which contributes to the excess value of medical equipment imports. Thus, the development of domestically produced surgical robots has long been awaited.

The hinotori™ Surgical Robot System (Medicaroid Corporation, Kobe, Japan) was the first Japanese-made surgical robot. Historically, Japan has produced many excellent industrial robots. Kawasaki Heavy Industries (Kobe, Japan), one of the largest manufacturers, has been involved in the development of the hinotori™ Surgical Robot System. Since its launch in 2020, more than 600 urological surgeries have been performed in Japan, and the safety of the initial results has been reported [1].

Robotic surgery for rectal cancer was first reported in 2006 [2] and has since been developed worldwide. Robotic surgery, featuring multi-joint function and 3D high-resolution images, is suitable for rectal surgery requiring narrow pelvic manipulation. Furthermore, it is expected to improve outcomes in terms of safety, curability, and function preservation. However, the results of the ROLARR trial, the first randomized controlled trial (RCT) comparing robotic and laparoscopic surgery in 2017, failed to show the superiority of robotic surgery [3]. Subsequently, more reports have emerged indicating that robotic surgery is significantly better in terms of short-term outcomes and oncological outcomes, gradually proving the usefulness of robotic surgery [4, 5]. In Japan, the VIRUVIANO trial is underway to examine the outcomes of robotic surgery at advanced medical centers, and the results will soon be published. In addition, various types of surgical robots, including the hinotori™ Surgical Robot System, have been introduced and are currently in operation. It is expected that new evidence will be developed and treatment outcomes will be further improved.

In 2022, the hinotori™ Surgical Robot System was approved in Japan for use in the gastrointestinal field, which is a huge market with a larger number of surgeries. Herein, we report the world’s first surgery for rectal cancer using the hinotori™ Surgical Robot System.

## Case presentation

**Fig. 1:**
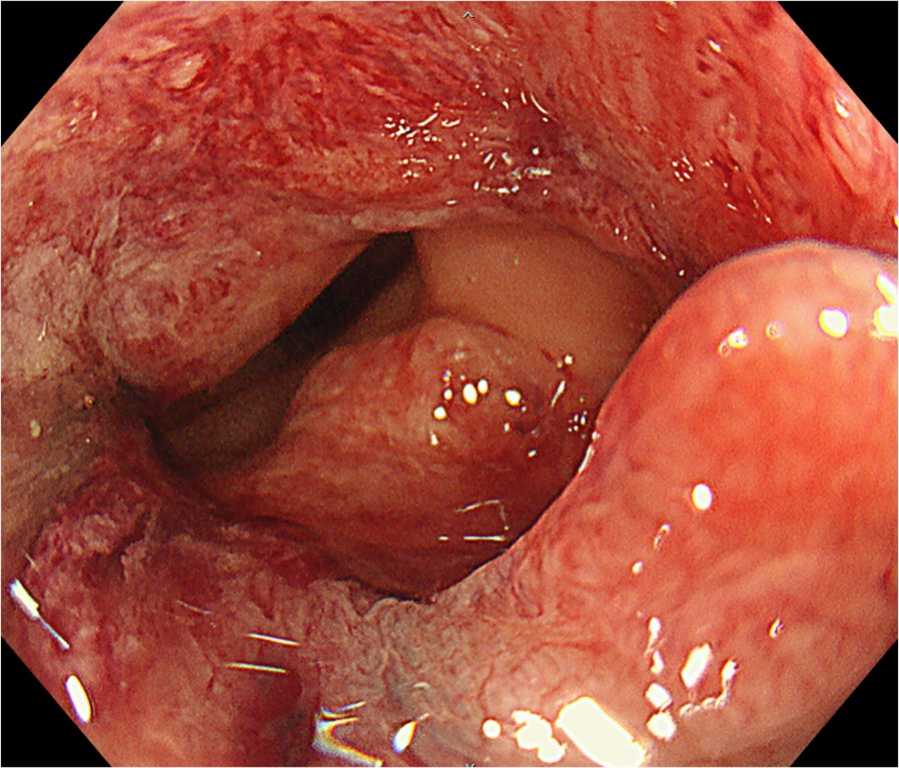
Pre-operative colonoscopy examination. Colonoscopy reveals a type 2 tumor extending to almost the entire circumference of the rectum

**Fig. 2:**
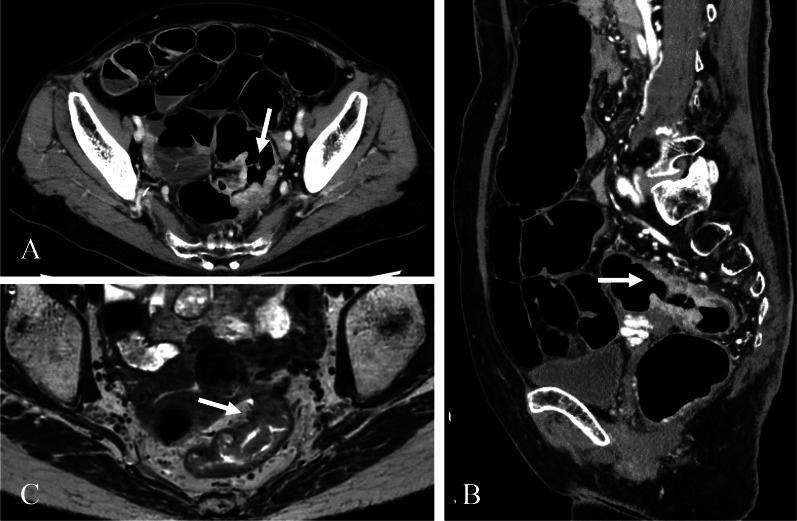
Pre-operative enhanced CT and MRI. **A**, **B** CT discloses wall thickening in the rectum (arrow). There are no enlarged lymph nodes or distant metastases (**A**: axial, **B**: sagittal). **C** MRI reveals tumor invasion beyond the intrinsic muscle layer of the rectum (arrow)

A 71-year-old woman with a body mass index (BMI) of 18.3 kg/m^2^ presented with bloody stools. The attending physician suspected rectal cancer and the patient was referred to our hospital for further investigation. A colonoscopy revealed type 2 advanced cancer in the rectum, 12 cm from the anal verge and 10 cm from the dentate line (Fig. 1). Histological examination disclosed a well-differentiated adenocarcinoma. Abdominal enhanced computed tomography (CT) revealed wall thickening in the rectum without significant swelling of the lymph nodes or distant metastasis. Pelvic magnetic resonance imaging (MRI) revealed tumor invasion beyond the intrinsic rectal muscle layer (Fig. 2). The patient was diagnosed with cStage IIA (cT3N0M0) rectal cancer based on the Japanese Classification of Colorectal, Appendiceal, and Anal Carcinoma: 3d English Edition Treatment with low anterior resection (LAR) was planned using the hinotori™ Surgical Robot System.

Colorectal cancer surgery with the hinotori™ Surgical Robot System was approved by Evaluating Committee for Highly Difficult New Medical Technologies (approval number 22-007) as well as the Institutional Review Board at Sapporo Medical University.

The surgeon (I.T.) had extensive experience in robotic surgery, underwent training as the first operator, as defined by a developing company (Medicaroid Corporation), and was certified by the Japan Society for Endoscopic Surgery.

**Fig. 3:**
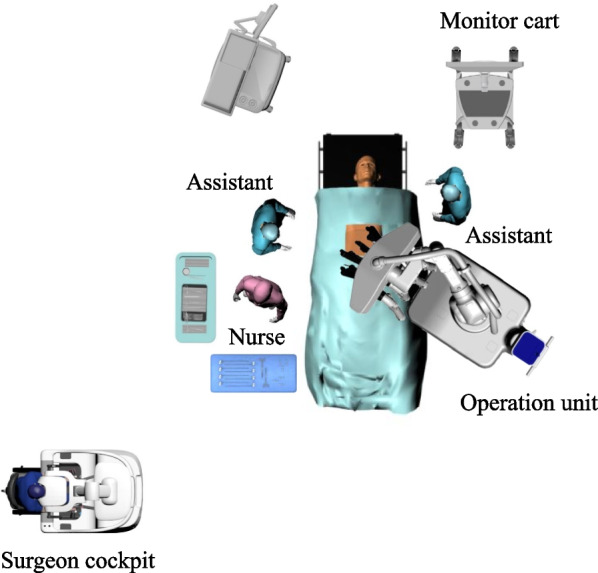
Layout of the surgical instruments. The operating unit is rolled in from the left side of the patient and the tilt setting of the arm base is 6° to the lower right

**Fig. 4:**
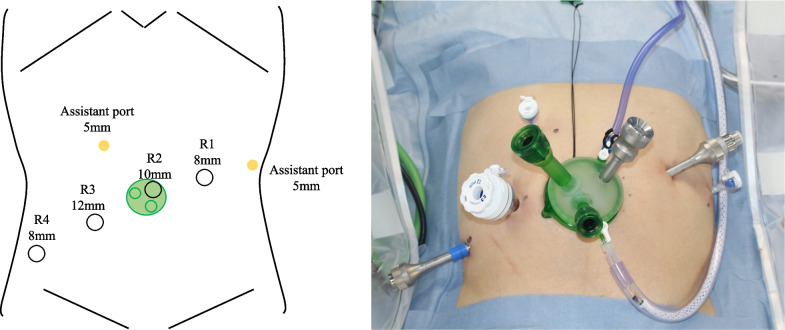
Port placement. A GelPOINT Mini (Applied Medical) is positioned at the umbilical site. R1–4 robotic arms; R1, R4 8 mm, R3 12 mm; and R2 port for the scope, 10 mm. Two assistant ports are placed on the upper right and lateral left sides of the abdomen

The layout of the surgical instruments is shown in Fig. 3. After a vertical skin incision of 3 cm was made in the umbilicus, a GelPOINT Mini (Applied Medical, Rancho Santa Margarita, CA, USA) was fitted into the incision, followed by the insertion of an assistant port. Four robotic ports and an additional assistant port were arranged as depicted in Fig. 4. The assistant port on the left side is used to assist in the deployment of the surgical field and to aspirate mist, and is also placed in the same manner when the da Vinci Surgical System is used in our department. The patient was positioned with the head lowered by 15° lower and the right lowered by 12°. The small intestine was positioned on the right cephalic side to secure the operative field in the abdominal cavity. The operation unit was rolled from the left side of the patient, and the tilt setting of the arm base was 6° to the lower right which was in line with the tilted body position of the patient. The instruments used in this surgery were bipolar fenestrated forceps for the robotic first arm, a scope for the second arm, monopolar curved scissors or bipolar Maryland forceps for the third arm, and Croce grasping forceps for the fourth arm.

**Fig. 5:**
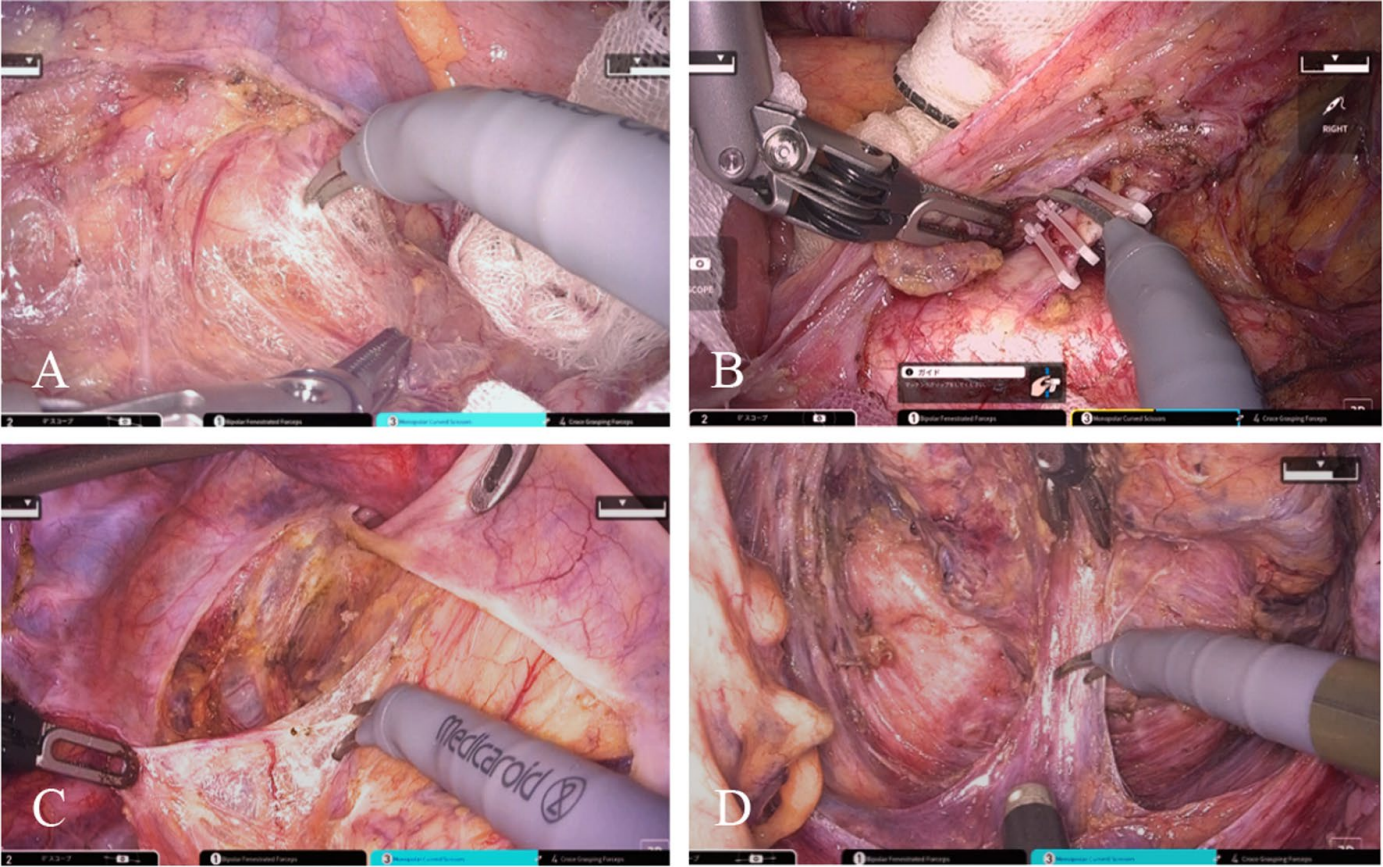
Intra-operative findings during robotic low anterior resection. **A** The left colon is dissected using a medial approach. **B** Lymph nodes around the IMA are dissected and the root of the IMA is ligated. **C** The right side of the rectum is dissected. **D** The posterior rectum is dissected

**Fig. 6:**
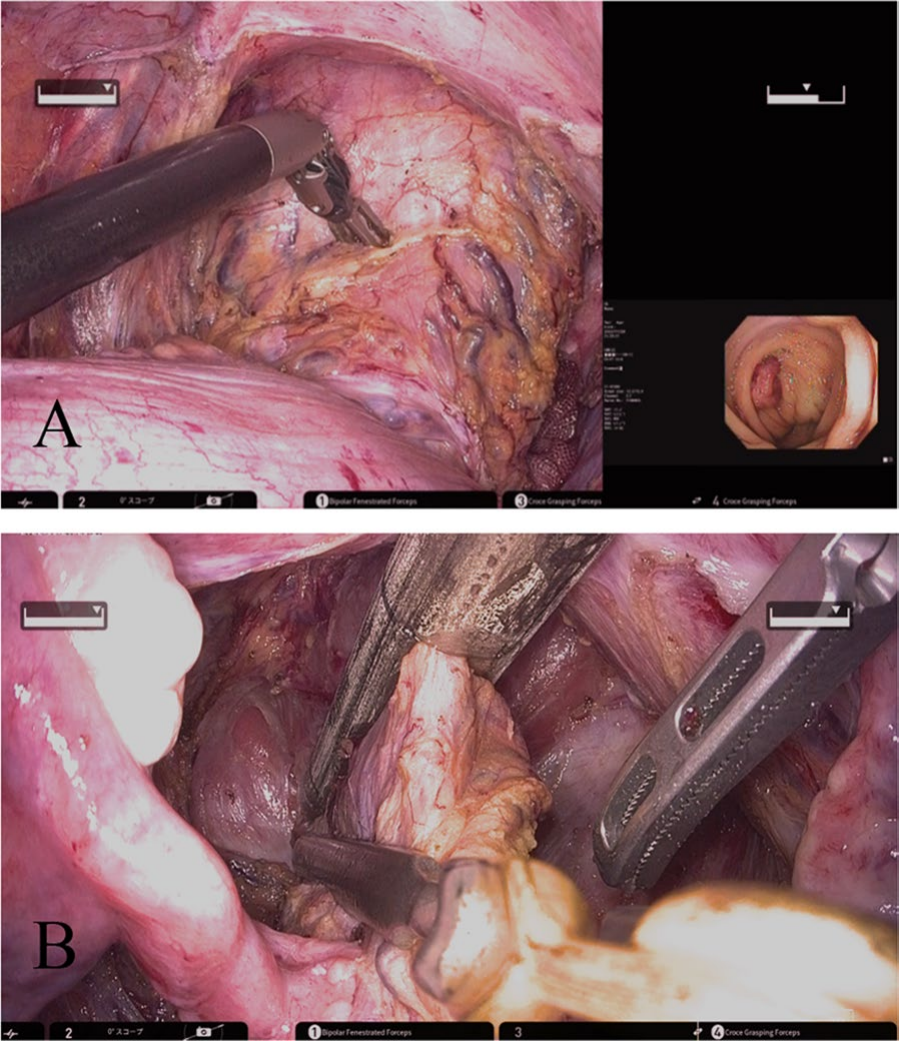
Intra-operative findings at the time of rectal transection. **A** The tumor is located endoscopically. **B** The rectum is dissected by the suturing device

The surgical procedures in the cockpit were performed in the same manner as those in the da Vinci Surgical System. The LAR was performed using the medial approach, preserving the inferior abdominal nerves. The root of the inferior mesenteric artery (IMA) was identified, surrounding lymph nodes were dissected, and the vessel was clipped and ligated (Fig. 5). The inferior mesenteric vein (IMV) was observed on the left side of the IMA and ligated. The rectum was dissected along the mesorectum, and all pelvic nerves were preserved. The tumor was located endoscopically, the distal margin was secured, tumor-specific mesorectal excision (TSME) was achieved, and the rectum was dissected using a suturing device (Fig. 6). After removing the operation unit, the rectal section was pulled out through the umbilical wound, the colon on the oral side of the tumor was ligated, and the specimen was excised. The intestine was anastomosed using the double-stapling technique. The operating time was 262 min, with a cockpit time of 134 min. Subsequently, the patient was discharged 10 days postoperatively without complications. No dysuria or other sequelae were observed. The pathological diagnosis was pStage IIA (cT3N0M0), with a circumferential resection margin (CRM), which was 6 mm.

## Discussion

The hinotori™ Surgical Robot System has several features that differ from those of the da Vinci Surgical System. First, each robotic arm has eight axes and the ability to set an arm base angle. This may be advantageous for rectal cancer surgery, which requires a wide operating range and is prone to arm interference. Arm interference is concerning as it may lead to operational difficulties and increase the risk of injury to other organs. In this case, we performed surgery with the arm base inclination set at 6° after pre-operative simulation using a model, and the operation was completed without any intra-operative difficulties. However, this surgical method is in the early stages of implementation, and determining the optimal settings remains a challenge. Second, the cockpit of the surgeon was designed using ergonomic methods, such as a flexible three-dimensional viewer. Reports indicate that robotic surgery reduces the perceptual and physical burden on the surgeon compared to open and laparoscopic surgery [6]. Moreover, the hinotori™ Surgical Robot System is expected to further reduce the burden on the surgeon.

However, some issues have emerged compared with the da Vinci Surgical System, which has been repeatedly updated and improved. These include issues related to operability, such as resonance between arms because the arms are connected, excessive operation stoppage owing to safety device sensing, and slow device startup. These limitations may prevent the operation as the surgeon had envisioned. Second, there is a lack of varied instrument types. Furthermore, there are no dedicated vessel-sealing devices or staplers at present. Although these devices may substitute laparoscopic devices, they are essential for smooth surgical progress and require further development. These features may be improved and should be overcome with future updates. Furthermore, the multi-jointed movements of the operation arm and the operation methods of the hand control or the foot unit are similar to those of the da Vinci Surgical System, and surgeons familiar with the da Vinci Surgical System will be able to achieve a smooth introduction and equivalent surgical procedures.

This case was the world's first LAR using the hinotori™ Surgical Robot System, with a complete TSME and negative CRM, without curative problems. The standard for rectal cancer surgery is to complete total mesorectal excision (TME) or TSME and ensure a CRM of at least 1 mm. Failure of this increases the risk of local recurrence [7]. However, incisions outside of the rectum increase the risk of pelvic nerve injury and postoperative dysfunction. In summary, rectal cancer surgery requires a delicate procedure to maintain an appropriate layer of dissection. Conventional laparoscopic surgery for rectal cancer failed to demonstrate non-inferiority in oncologic outcomes compared with open surgery in several RCTs (COLOR II, COREAN, ACOSOG Z6051, and ALaCaRT) [8–11]. In the PRODUCT trial conducted at an advanced center in Japan, the CRM positivity rate was 8.6%, which is similar to the results of other RCTs [12]. One possible reason for the lack of good results is that restrictions owing to linear forceps manipulation in the deep pelvis may make it challenging to secure the CRM. Robotic surgery, which is more functionally advantageous, may overcome these limitations. The first RCT, the ROLARR trial, showed no significant difference in CRM-positive rates compared with laparoscopy (5.1% vs. 6.3%) [3] however, in the latest REAL trial, robotic surgery was significantly better (4.0% vs. 7.2%) [5]. The oncological results of surgery using the hinotori™ Surgical Robot System will be accumulated in the future.

The operation time was found to be approximately 30 min longer than that of LAR using the da Vinci Surgical System when performed by the same surgeon. The main reasons for this are that this was the first case of this kind, so safety was important, and there remain issues in terms of operability to be resolved. In addition, the learning curve for robotic surgery for rectal cancer has been reported to reach proficiency in 12–36 cases [13–15]. With more cases, a reduction in the operative time was achieved.

The patient had an uneventful clinical course without postoperative complications. Robotic surgery has been reported to have better short-term outcomes than laparoscopic surgery. In the REAL trial, the complication rate of Clavien–Dindo grade 2 or higher within 30 days after surgery was 16.2% vs. 23.1%, which was significantly better for robotic surgery than laparoscopic surgery [5]. In a study using the National Clinical Database in Japan, intra-operative blood loss was significantly lower (15 ml vs. 20 ml), and the postoperative hospital stay was significantly shorter (13 days vs. 14 days) in robotic surgery than in laparoscopic surgery [4]. Additionally, a meta-analysis suggested that robotic surgery is associated with fewer postoperative urinary dysfunctions and is useful for preserving function [16]. Similar or better outcomes may be expected from surgery using the hinotori™ Surgical Robot System and we have already started a prospective study on treatment outcomes.

Several more surgical robots are currently under development, and their use will be concerning in the future. It is desirable to select a robot with the most appropriate functions and advantages for each case and it is necessary to create a foundation for this purpose. The emergence of new surgical robots will further develop robotic surgery by creating appropriate market competition, advancing functionality, and lowering costs.

## Conclusions

We report the first case of LAR for rectal cancer using the hinotori™ Surgical Robot System by which a safe and oncologically appropriate surgery was performed.

## Data Availability

The dataset supporting the conclusions of this article is included within the article.

## Abbreviations

RCT: Randomized controlled trial
BMI: Body mass index
CT: Computed tomography
MRI: Magnetic resonance imaging
LAR: Low anterior resection
IMA: Inferior mesenteric artery
IMV: Inferior mesenteric vein
TSME: Tumor-specific mesorectal excision
CRM: Circumferential resection margin
TME: Total mesorectal excision
